# Successful implantation of leadless pacemakers in children: a case series

**DOI:** 10.1093/ehjcr/ytaa064

**Published:** 2020-03-30

**Authors:** Ewa Jędrzejczyk-Patej, Aleksandra Woźniak, Linda Litwin, Alina Skiba-Zdrzałek, Michał Mazurek, Radosław Lenarczyk, Zbigniew Kalarus, Oskar Kowalski

**Affiliations:** y1 Department of Cardiology, Congenital Heart Diseases and Electrotherapy, Silesian Centre for Heart Diseases, Skłodowskiej-Curie 9, 41-800 Zabrze, Poland; y2 School of Medicine with the Division of Dentistry, Zabrze, Medical University of Silesia, Katowice, Poland; y3 Department of Congenital Heart Diseases and Pediatric Cardiology, Silesian Center for Heart Diseases, Skłodowskiej-Curie 9, 41-800 Zabrze, Poland; y4 Department of Cardiology, Silesian Center for Heart Diseases, Skłodowskiej-Curie 9, 41-800 Zabrze, Poland; y5 Department of Dietetics, School of Public Health in Bytom, Medical University of Silesia, Poniatowskiego 15, 40-055 Katowice, Poland

**Keywords:** Leadless pacemaker, Micra, Pacing, Children, Case series

## Abstract

**Background:**

A leadless pacemaker is a new concept in which a miniaturized pacing device is self-contained within the heart. Recently published data show that leadless pacemakers are associated with a decreased risk of major complications when compared with transvenous cardiac pacemakers. This seems to be of particular importance in children and young adults in whom various complications may occur during their lifetime.

**Case summary:**

Herein, we report the successful implantation of Micra™ Transcatheter Pacing System in two children: 12-year-old boy and 13-year-old girl, along with a long-term follow-up. The children had indications for pacemaker implantation, however, with an expected low percentage of pacing due to paroxysmal nature of the third-degree atrioventricular block. The implantation procedures were performed in general anaesthesia. There were no complications. During the 2-year follow-up, there were no adverse events and the electrical parameters of the device remained stable. Pacing percentage was below 0.1%.

**Discussion:**

Transvenous cardiac pacemakers improve quality of life and reduce mortality but may be associated with various short- and long-term complications, mainly related to the presence of transvenous leads and the pulse generator. Compared with adult patients, the implantation of conventional pacemakers in children is still a challenge, not only because of their smaller size but also due to continuing growth, as well as a higher rate of lead and device-related complications. We demonstrate that the implantation of leadless pacemakers in children is feasible and could be worth considering in certain clinical scenarios, especially when ventricular pacing is required rarely.

**Learning points**

- A leadless pacemaker is a new miniaturized pacing device which allows to eliminate potential complications associated with the presence of transvenous leads and the pulse generator.
- Our case series shows that the implantation of leadless pacemakers in children is feasible and could be the worth considering solution when ventricular pacing is required rarely. During the 3-year follow-up, there were no adverse events and the electrical parameters of the leadless pacemaker in our paediatric patients remained stable.

## Introduction

Transvenous cardiac pacemakers not only significantly improve quality of life but also reduce mortality. Unfortunately, despite the substantial progress achieved throughout decades in the pacing technology, conventional cardiac pacemakers are still associated with various short- and long-term complications mainly related to transvenous leads.^1^

A leadless pacemaker is a new concept in which a miniaturized pacing device is self-contained within the heart. This technology allows to eliminate potential complications associated with the presence of transvenous leads and the pulse generator. The safety and clinical performance of transcatheter leadless pacemakers have previously been assessed in clinical trials.^2^^,^^3^ Clinical trial experience with the Micra™ Transcatheter Pacing System (Micra TPS, Medtronic, Minneapolis, MN, USA) began in 2013, whereas it received CE mark in April 2015 and FDA approval in April 2016. According to the published data, the implant success rate of Micra™ in adults is approximately 99% and this technology is associated with almost 50% fewer major complications when compared with conventional pacemakers.^4^^,^^5^ We report one of the first in the world successful implantation of Micra™ TPS in two children and their further long-term follow-up.

## Timeline

**Table:** 

Time	Events	
	Patient 1	Patient 2
Before admission	Three episodes of syncope. Highly symptomatic third-degree atrioventricular (AV) block was diagnosed in the patient.	Permanent first-degree AV block. An episode of asymptomatic paroxysmal third-degree AV block with a 6-s pause registered in ambulatory electrocardiogram Holter.
During the admission	The physical examination revealed no other abnormalities	
Procedure	Implantation of the Micra™ Transcatheter Pacing System without complications	
After the implantation	No complications	
	Discharged home on the 14th day post-implantation	Discharged 7 days after the procedure
Follow-up	The 32-month follow-up: no adverse events, no recurrences of syncope, and proper leadless pacemaker function	The 37-month follow-up: no adverse events and stable electrical parameters of the device

## Case presentation

### Patient 1

A 12-year-old boy who underwent surgical repair of congenital heart defect (atrial septal defect type II and ventricular septal defect) at the age of 3 months was urgently transferred from a regional hospital to our centre due to highly symptomatic third-degree atrioventricular (AV) block. The patient was brought by ambulance after three episodes of syncope with seizures lasting for 2 min followed by spontaneous recovery of consciousness. Both the neurological examination and computed tomography of the head revealed no abnormalities. Due to the recurrence of complete AV block with significant bradycardia and hypotension, temporary transvenous cardiac pacing was initiated in the referring hospital.

On admission, the patient was drowsy, but conscious, without any neurological deficits, with a heart rate of 85/min (temporary pacing in the VVI mode) and blood pressure of 115/42 mmHg. The physical examination revealed no other abnormalities except for a median sternotomy scar. The body weight and height of the boy were 45 kg and 140 cm, respectively. A 12-lead electrocardiogram (ECG) showed VVI pacing, however, when the pacing was temporarily turned off there was a sinus rhythm 60–70/min with 1:1 AV conduction. The transthoracic echocardiography showed normal chamber dimensions, good systolic function of both ventricles (left ventricular ejection fraction of 78%), mild tricuspid regurgitation, and no residual shunting through interatrial and interventricular septum. Laboratory results were within normal ranges: white blood cells of 8.9 × 10^3^/µL, red blood cells of 5.85 × 10^6^/µL, haemoglobin—8.2 mmol/L, creatinine—55 µmol/L, potassium—4.70 mmol/L, and C-reactive protein—2.71 mg/L.

During the follow-up since 2003, asymptomatic episodes of both first- and second-degree paroxysmal AV block (2:1) have already been observed, however, parents repeatedly refused pacemaker implantation and the boy was treated with metaproterenol with a good dromotropic effect remaining asymptomatic over the years (paroxysmal high degree AV block was occasionally present when the heart rate exceeded 140/min; the older the patient got, the slower the heart rate was and the fewer AV block episodes were registered). Regardless of the past, the child now had strong and unquestioned indications for pacemaker implantation, however, with an expected low percentage of right ventricular (RV) pacing due to previously asymptomatic follow-up and paroxysmal nature of the AV block. Both, parents and the boy repeatedly expressed their psychological resistance to a traditional device. They were afraid of the cosmetic effect of the implant, various restrictions related to traditional pacemaker and long-term device-related complications. Having considered the overall clinical situation and having thoroughly discussed the issue with the parents, who continuously showed considerable reluctance to conventional pacemaker implantation, the consulting electrophysiologist decided to implant a single chamber leadless pacemaker Micra™. A written informed consent was obtained from parents prior to the procedure.

**Figure 1:**
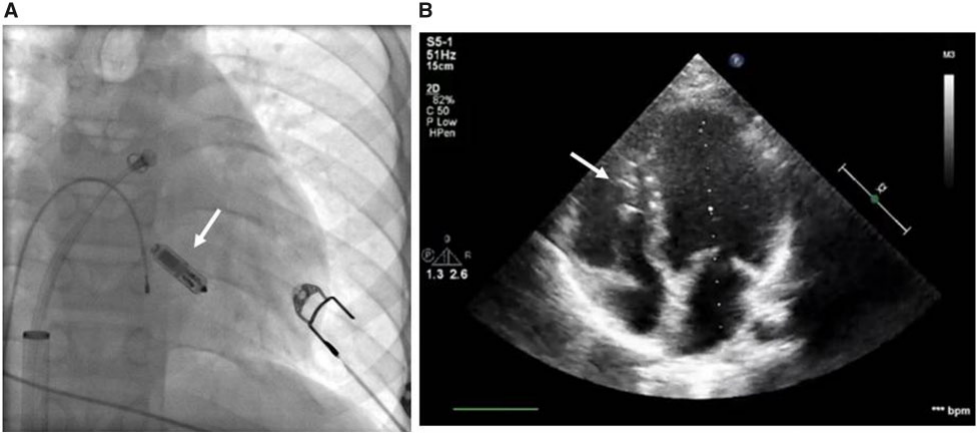
Location of the Micra™ device in fluoroscopy during the implantation procedure (*A*) and in transthoracic echocardiography (*B*) in a Patient 1. Location is marked with white arrows.

The implantation procedure was performed in general anaesthesia on February 2016. A dedicated Transfemoral Delivery Catheter was used to advance the device into the right ventricle through the 23-Fr Micra Introducer placed in the right femoral vein. We performed a predilatation with 8-Fr, 12-Fr, and 18-Fr sheaths, receptively, followed by a proper 23-Fr introducer sheath insertion. At the time of 8-Fr femoral sheath use, we also performed angiography of femoral vein. Micra™ was attached to the middle part of the intraventricular septum with two out of four self-expanding nitinol tines (*Figure 1*). Appropriate and stable device fixation was achieved after the first deployment and was assessed by the following: fluoroscopic visualization, pull and hold test, tactile feel, and appropriate electrical measurements. Having confirmed proper device implantation, the tether was cut, the delivery system was removed and the insertion site was closed with a haemostatic suture. Total fluoroscopy and procedure duration was 3 min 7 s and 55 min, whereas the fluoroscopy dose and dose area product were 64 mGy and 378.6 µGym^2^, respectively. There were no peri- or post-procedural complications. The location of the device is shown in *Figure 1*.

**Figure 2:**
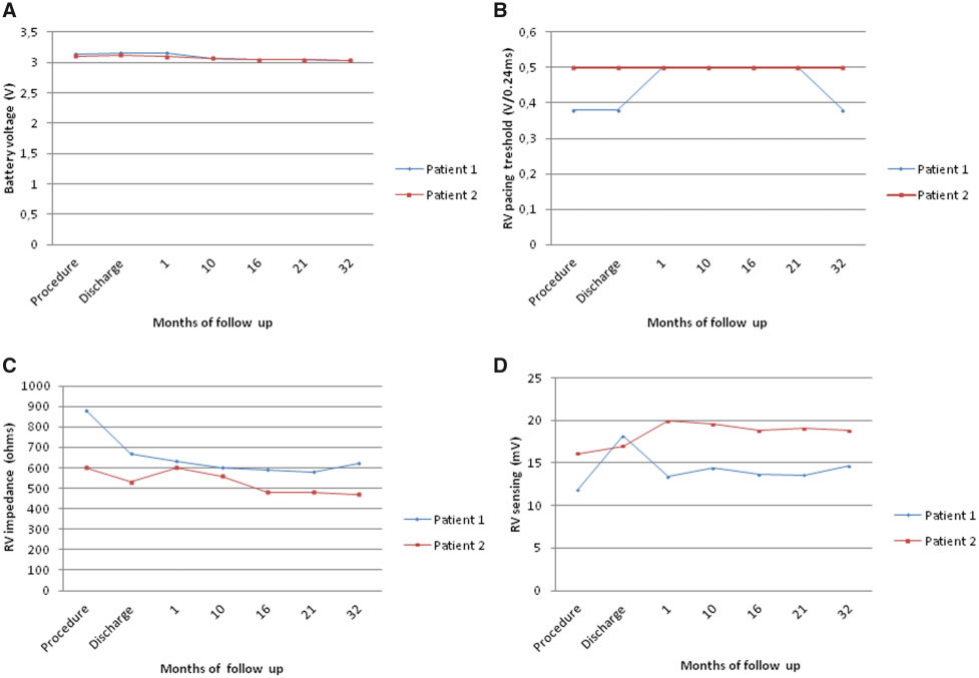
The electrical parameters of the device: battery voltage (*A*), right ventricular pacing threshold (*B*), right ventricular impedance (*C*), and right ventricular sensing (*D*). RV, right ventricle.

In the early post-operative period, the high-degree AV block prevailed with a high percentage of RV pacing (initially around 50%). The boy was discharged home on the 14th day post-implantation with 65% of intrinsic AV conduction and Micra™ programmed to VVI 40/min. Since then, the child has been carefully observed in the cardiology outpatient clinic. During the 32-month follow-up, there were no adverse events or recurrences of either pre- or syncope with the proper device function and significant reduction of RV pacing to less than 0.1%. The electrical parameters of the device have been presented in *Figure 2*.

### Patient 2

A 13-year-old girl with permanent first-degree AV block diagnosed at the age of 10 during routine pre-participation screening for sport activity was admitted to the same department 2 months later. The reason for admission was an episode of asymptomatic paroxysmal third-degree AV block with a 6-s pause registered in one of ambulatory ECG Holter recordings performed routinely in the last 3 years.

The development of the girl was normal without any episodes of presyncope or syncope in the past. The physical examination revealed no abnormalities except for a silent systolic murmur heard at the Erb’s point. The body weight and height of the girl were 60 kg and 156 cm, respectively. The 12-lead ECG demonstrated no other abnormalities except for the first-degree AV block (PR interval of 220 ms). Transthoracic echocardiography revealed no structural heart disease, the left ventricular ejection fraction was 66%. The girl showed good exercise tolerance, with a normal blood pressure response, appropriate chronotropic reaction and first-degree AV block throughout the exercise test. Laboratory blood results were within normal ranges: white blood cells of 9.3 × 10^3^/µL, red blood cells—4.8 × 10^6^/µL, potassium—4.7 mmol/L, and C-reactive protein—2.3 mg/L. Having excluded secondary causes of conduction abnormalities, the initial decision was made to perform conventional cardiac pacemaker implantation. However, parents refused the implantation of a traditional pacemaker. They would accept only the leadless device for their child. Of note, neither cardiology guidelines nor any expert consensus have been published with regard to leadless pacemakers. Thus, any decision on implantation of Micra has to be based only on previous papers reporting feasibility and safety of the method.^3^^,^^6^ The rationale behind this decision was the low likelihood for frequent RV pacing due to very sporadic episodes of asymptomatic complete AV block. A written informed consent was obtained from parents prior to the procedure and after a thorough discussion regarding all aspects of the device implantation and the potential need for pacemaker removal in the future.

**Figure 3:**
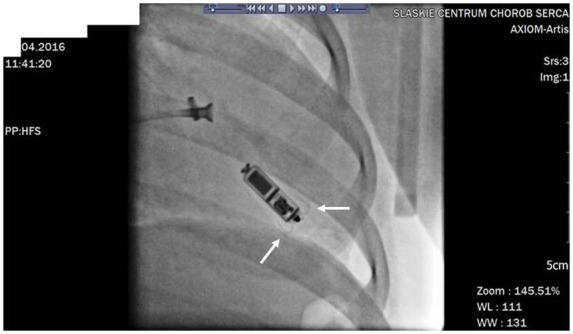
Micra™ implantation procedure in a Patient 2. Two self-expanding nitinol tines are marked with white arrows.

The implantation was performed in general anaesthesia on April 2016 using the same technique as described previously. Micra™ was attached to the inferior part of the intraventricular septum (*Figure 3*). Secure fixation to the myocardium was confirmed by intraprocedural measurements, fluoroscopic visualization, and the pull and hold test which showed that two nitinol tines were engaged (*Figure 3*). From the initial venous puncture to the closure of the insertion site, the procedure lasted 29 min. Total fluoroscopy duration was 2 min 5 s, while the fluoroscopy dose was 35 mGy (dose area product: 305.2 µGym^2^). After the procedure, the device was re-evaluated and programmed to VVI with the lower rate 45/min. No peri- or post-procedural complications were observed. Seven days after the procedure, the child was discharged from the hospital and has been followed up in the cardiology outpatient clinic since then. During the 37-month follow-up, there were no adverse events and the electrical parameters of the device remained stable (*Figure 2*). Right ventricular pacing percentage was below 0.1%. Estimated maximum and minimum longevity of the battery at the end of 37 months of follow-up were above 7 and 7 years, respectively.

## Discussion

Our case reports demonstrate that the implantation of leadless pacemakers is feasible in children. To the best of our knowledge, until now there has been published one case report on implantation of Micra™ in paediatric patient.^7^

Compared with older patients, the implantation of conventional pacemakers in children is still a challenge not only because of their smaller body sizes but also due to continuing growth and high level of patient activity as well as a higher rate of lead and device-related complications, for example lead fractures and dislodgements, venous occlusion and thrombosis, skin erosion at generator, tricuspid regurgitation, and so on. Previously published data showed that during mean 6 years of follow-up the incidence of lead complications in paediatric patients ranges between 7.2% and 23%.^8^^,^^9^ Five years survival rates of endocardial electrodes in children are lower than in adults.^10^ Recently published data showed that leadless pacemakers are associated with a decreased risk of major complications when compared with traditional devices.^4^^,^^5^ It seems to be particularly important in children and young patients in whom many different complications may occur during the lifetime. This new technology is very promising, but not free from limitations. At present, leadless pacemakers are available only as single chamber (ventricular) devices, thus patients in whom AV synchrony is not needed, such as those with atrial fibrillation, are potentially the best candidates for this technique. The VVI pacing mode in patients with sinus rhythm may cause symptoms of pacemaker syndrome, but single-chamber ventricular pacing might be functional if pacing is required very rarely (e.g. to prevent asystole during sporadic episodes of paroxysmal AV block or sinus arrest). Children, in whom atrial fibrillation is rather rare, but with sinus rhythm and infrequent episodes of paroxysmal high degree AV block, especially when a low percentage of RV pacing is expected, might be potentially good candidates for leadless pacemakers. Leadless pacing technology may protect the young subclavian venous system from thrombosis and occlusion and leave it patent for other medical procedures in the future. Moreover, due to the elimination of intravascular leads and miniaturization of the device, this technique prevents from multiple pocket and lead-related complications, including lead-induced tricuspid regurgitation which is associated with a poor prognosis.^11^

No data are so far available regarding removal of leadless pacemaker over time, for example after batter depletion. The manufacturer of the Micra pacemaker ensures that up to three devices may be safely placed within the right ventricle, without compromising their function. Taking into account current parameters of the pacemakers in both children, the batteries should allow for pacing on demand over the next 15 years. Following that period, a new device may be implanted (next to the previous one). Such an approach could be sufficient for a safe back-up VVI pacing over the next 30 years. The constantly evolving technology may possibly allow for increasingly safe and straightforward removal of such devices in the future or perhaps even percutaneous re-charging.

## Conclusions

We have demonstrated that the implantation of Micra™ TPS in children is possible and worth considering in certain clinical scenarios when ventricular pacing is required very rarely. However, further studies are needed to definitely assess feasibility and safety of leadless pacing technology in children in long-term observation.

## Lead author biography

**Figure:**
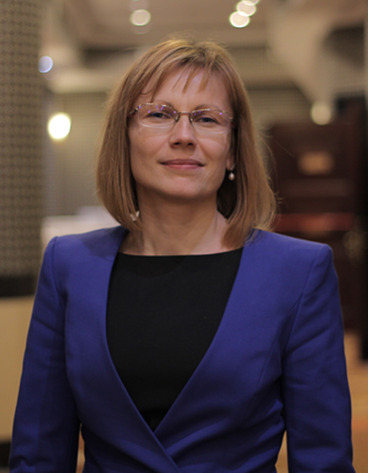


Ewa Jędrzejczyk-Patej is a cardiologist. She has graduated from the Medical University of Silesia in Katowice. She is working at the Department of Cardiology, Congenital Heart Diseases and Electrotherapy, Silesian Center for Heart Diseases in Zabrze since 2008. Her specialty is clinical electrophysiology. She is a Professional member of European Society of Cardiology and EHRA and member of Polish Cardiac Society and Polish Heart Rhythm Section.

## Supplementary material

Supplementary material is available at *European Heart Journal - Case Reports* online.

**Slide sets:** A fully edited slide set detailing this case and suitable for local presentation is available online as Supplementary data.

**Consent:** The author/s confirm that written consent for submission and publication of this case report including image(s) and associated text has been obtained from the patients in line with COPE guidance.

**Conflict of interest:** A.W.—consultant fees from Biotronik and Medtronic. E.J.-P., M.M., R.L., O.K.—consultant fees from Biotronik, Medtronic, Abbott and Boston Scientific. All other authors have declared no conflict of interest.

## Supplementary Material

ytaa064_Supplementary_Slide-Set
