# Supplementary material for: Successful implantation of leadless pacemakers in children: a case series
Source: Eur Heart J Case Rep. 2020 Mar 30;4(3):1–6. doi: 10.1093/ehjcr/ytaa064 (PMC7319807; doi:10.1093/ehjcr/ytaa064)
Supplement: ytaa064_Supplementary_Slide-Set [file ytaa064_supplementary_slide-set.pptx]

## Slide 1
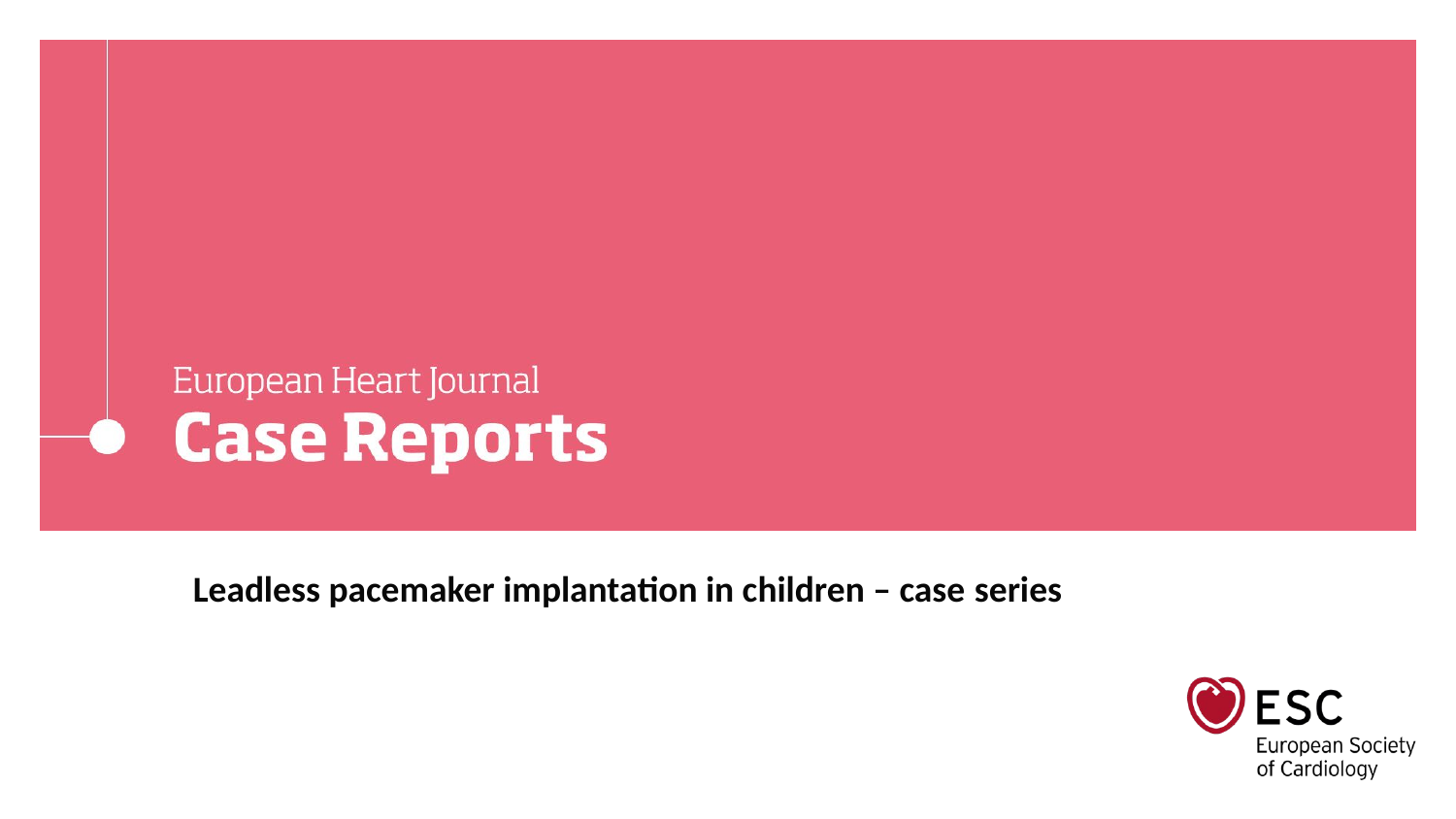

# Leadless pacemaker implantation in children – case series

## Slide 2
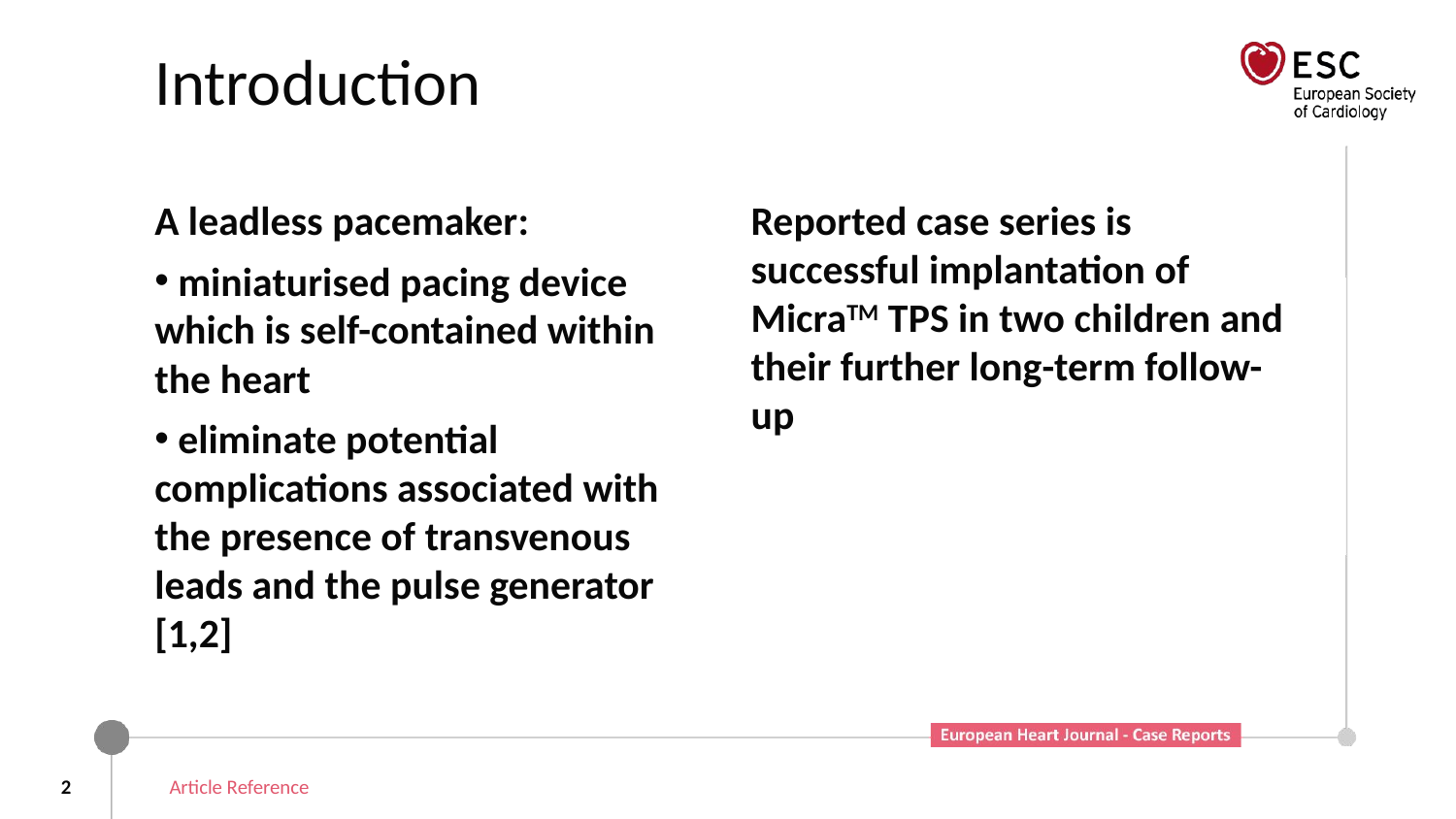

# Introduction
A leadless pacemaker:
 miniaturised pacing device which is self-contained within the heart
 eliminate potential complications associated with the presence of transvenous leads and the pulse generator [1,2]
Reported case series is successful implantation of MicraTM TPS in two children and their further long-term follow-up
2
Article Reference

## Slide 3
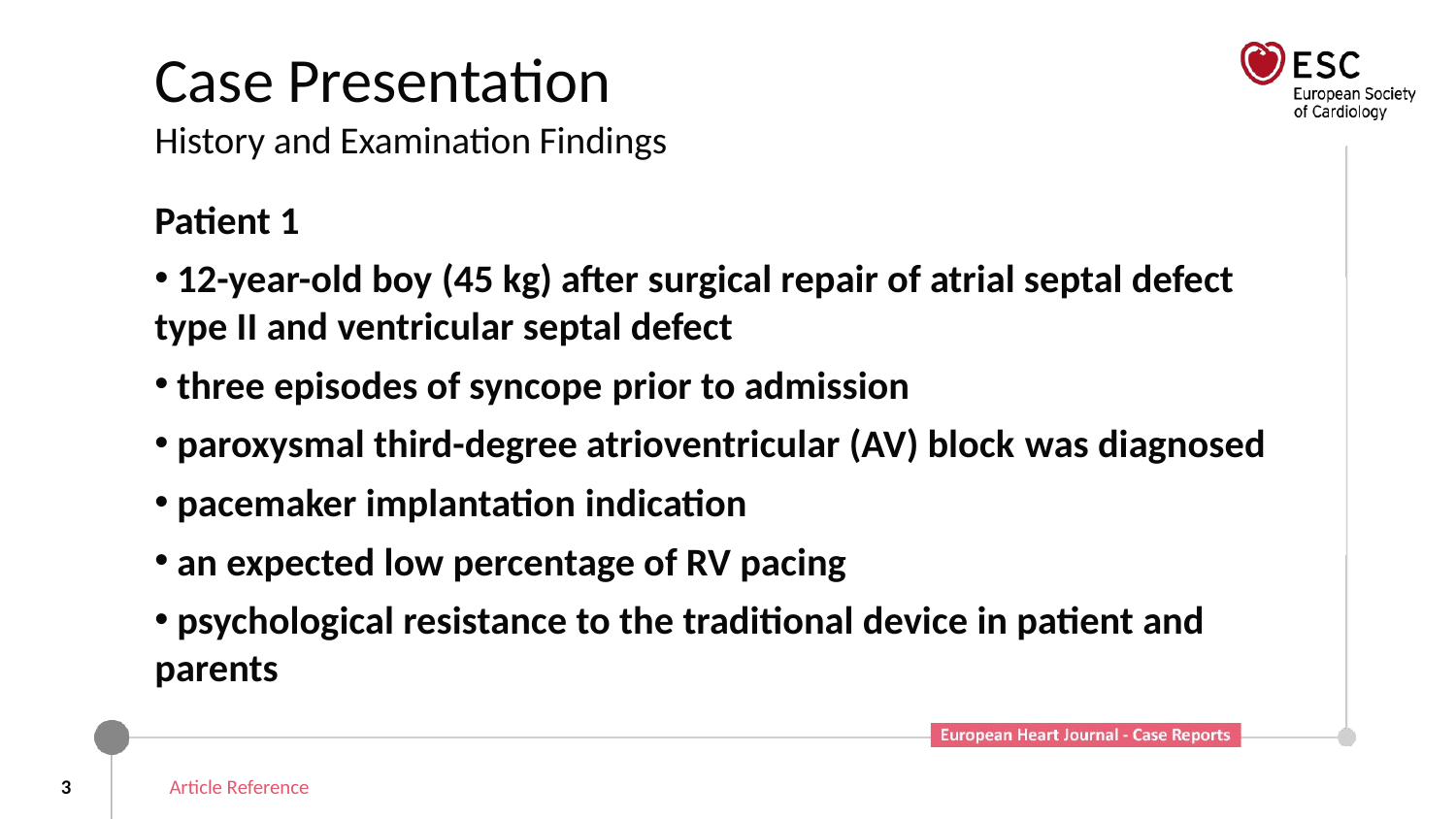

# Case Presentation History and Examination Findings
Patient 1
 12-year-old boy (45 kg) after surgical repair of atrial septal defect type II and ventricular septal defect
 three episodes of syncope prior to admission
 paroxysmal third-degree atrioventricular (AV) block was diagnosed
 pacemaker implantation indication
 an expected low percentage of RV pacing
 psychological resistance to the traditional device in patient and parents
3
Article Reference

## Slide 4
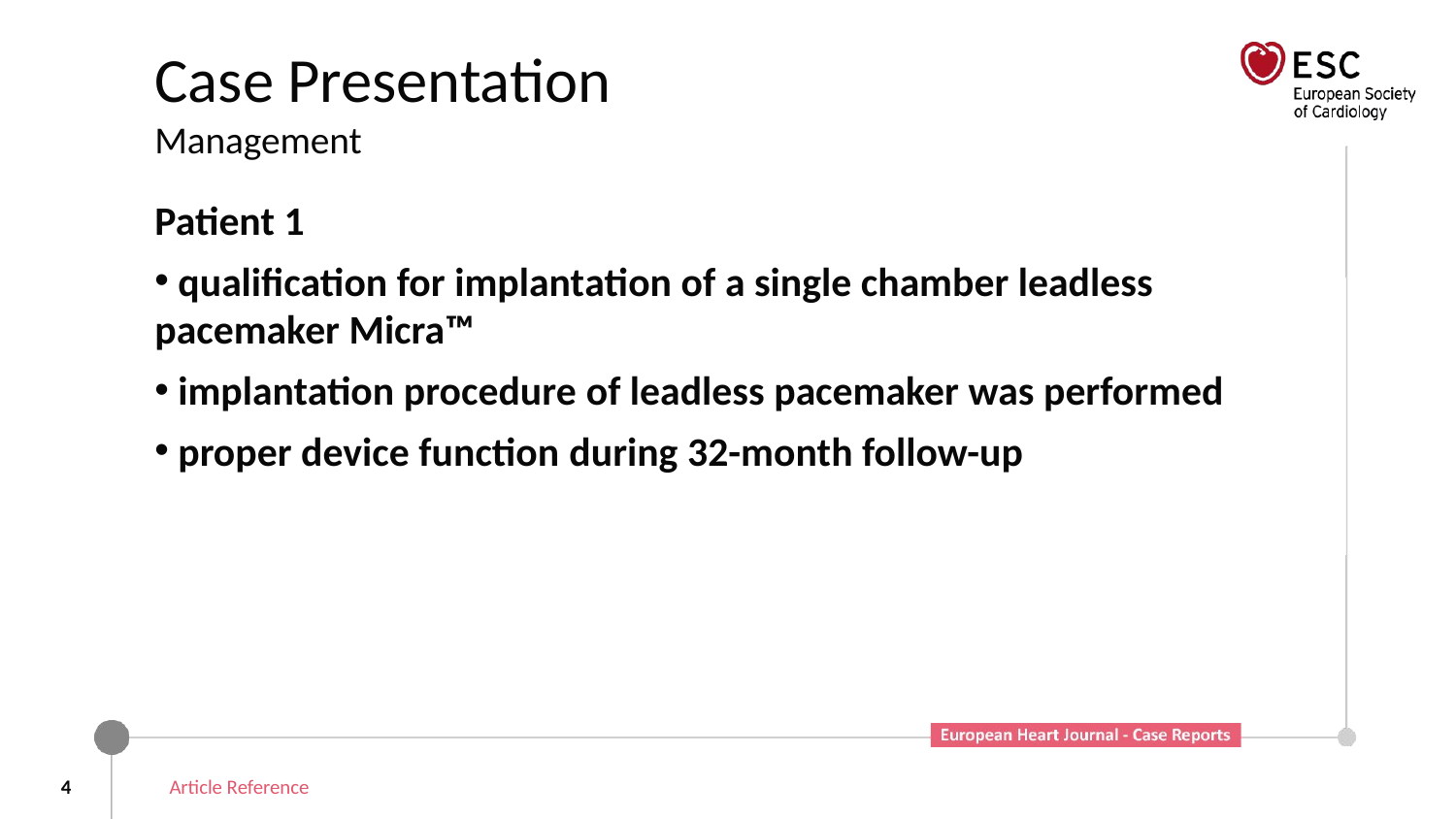

# Case Presentation Management
Patient 1
 qualification for implantation of a single chamber leadless pacemaker Micra™
 implantation procedure of leadless pacemaker was performed
 proper device function during 32-month follow-up
4
Article Reference

## Slide 5
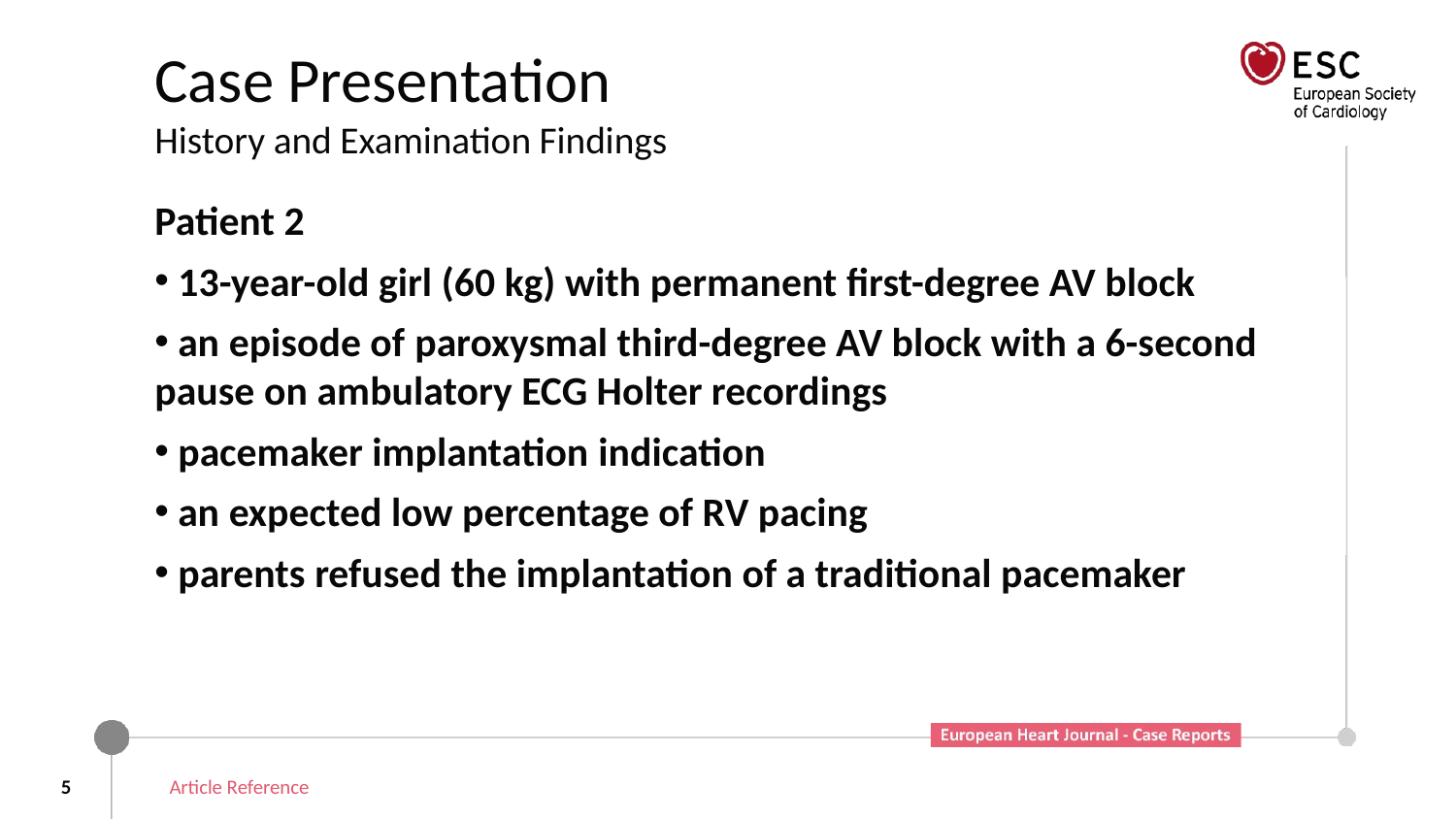

# Case Presentation History and Examination Findings
Patient 2
 13-year-old girl (60 kg) with permanent first-degree AV block
 an episode of paroxysmal third-degree AV block with a 6-second pause on ambulatory ECG Holter recordings
 pacemaker implantation indication
 an expected low percentage of RV pacing
 parents refused the implantation of a traditional pacemaker
5
Article Reference

## Slide 6
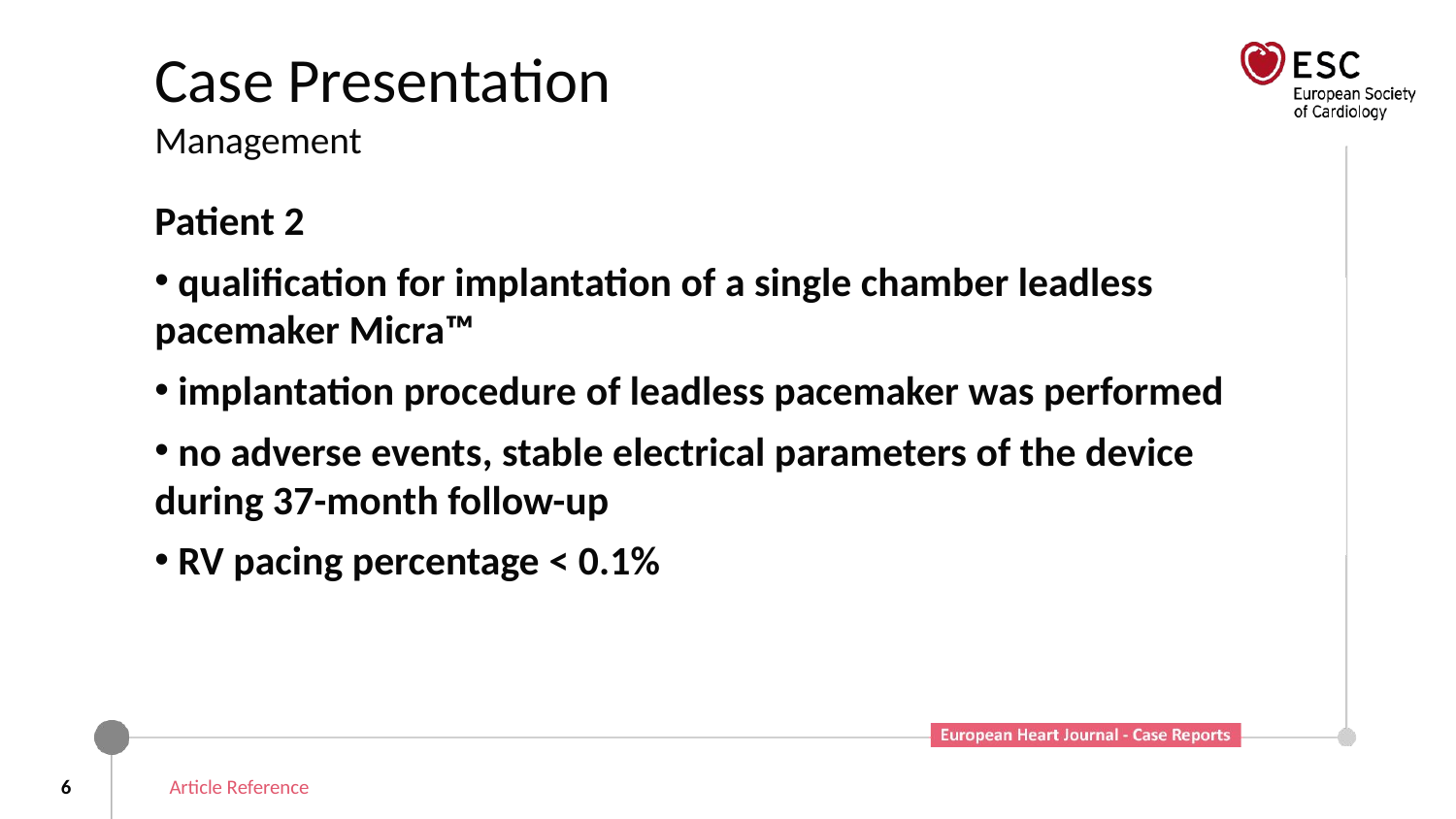

# Case Presentation Management
Patient 2
 qualification for implantation of a single chamber leadless pacemaker Micra™
 implantation procedure of leadless pacemaker was performed
 no adverse events, stable electrical parameters of the device during 37-month follow-up
 RV pacing percentage < 0.1%
6
Article Reference

## Slide 7
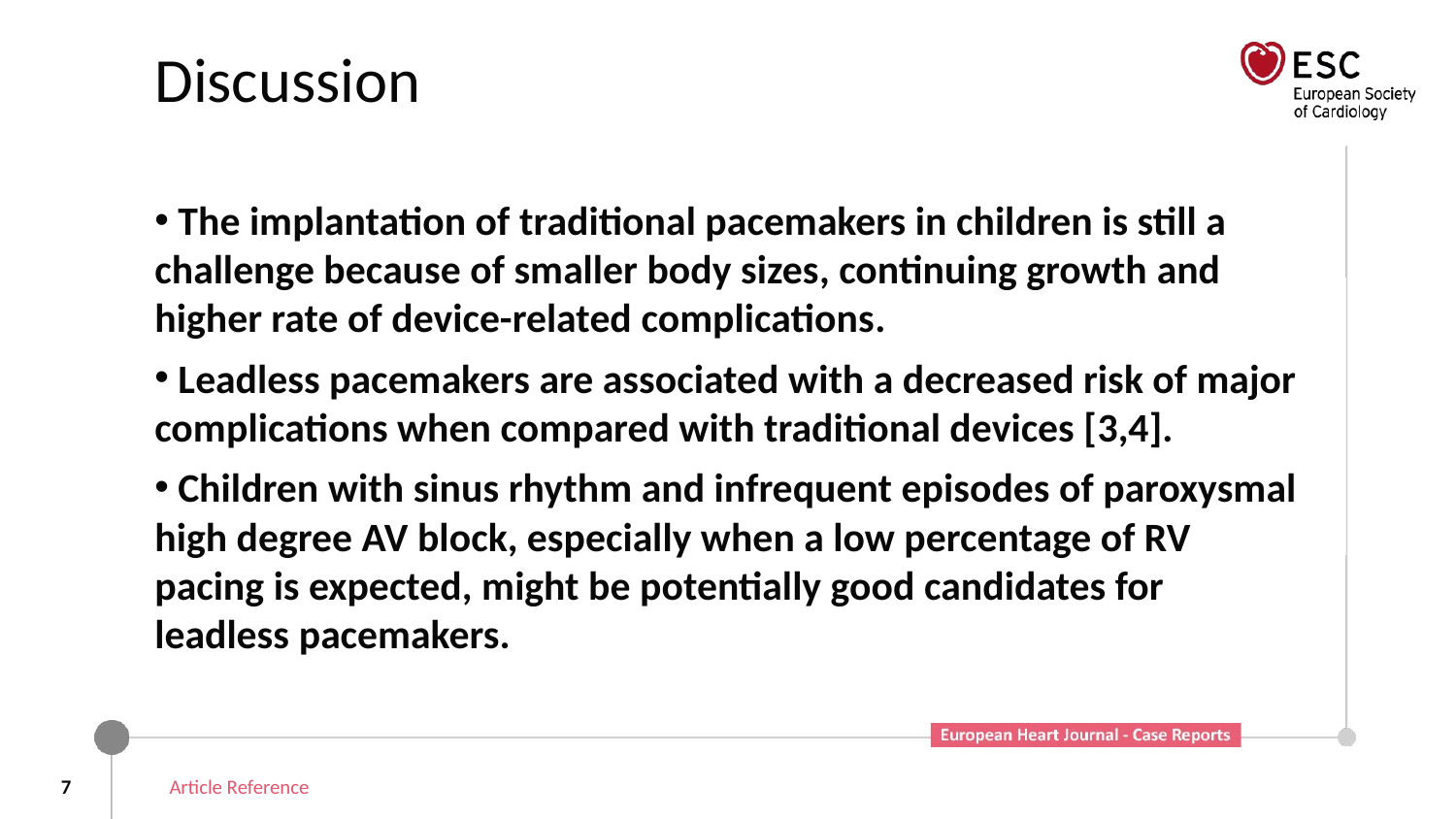

# Discussion
 The implantation of traditional pacemakers in children is still a challenge because of smaller body sizes, continuing growth and higher rate of device-related complications.
 Leadless pacemakers are associated with a decreased risk of major complications when compared with traditional devices [3,4].
 Children with sinus rhythm and infrequent episodes of paroxysmal high degree AV block, especially when a low percentage of RV pacing is expected, might be potentially good candidates for leadless pacemakers.
7
Article Reference

## Slide 8
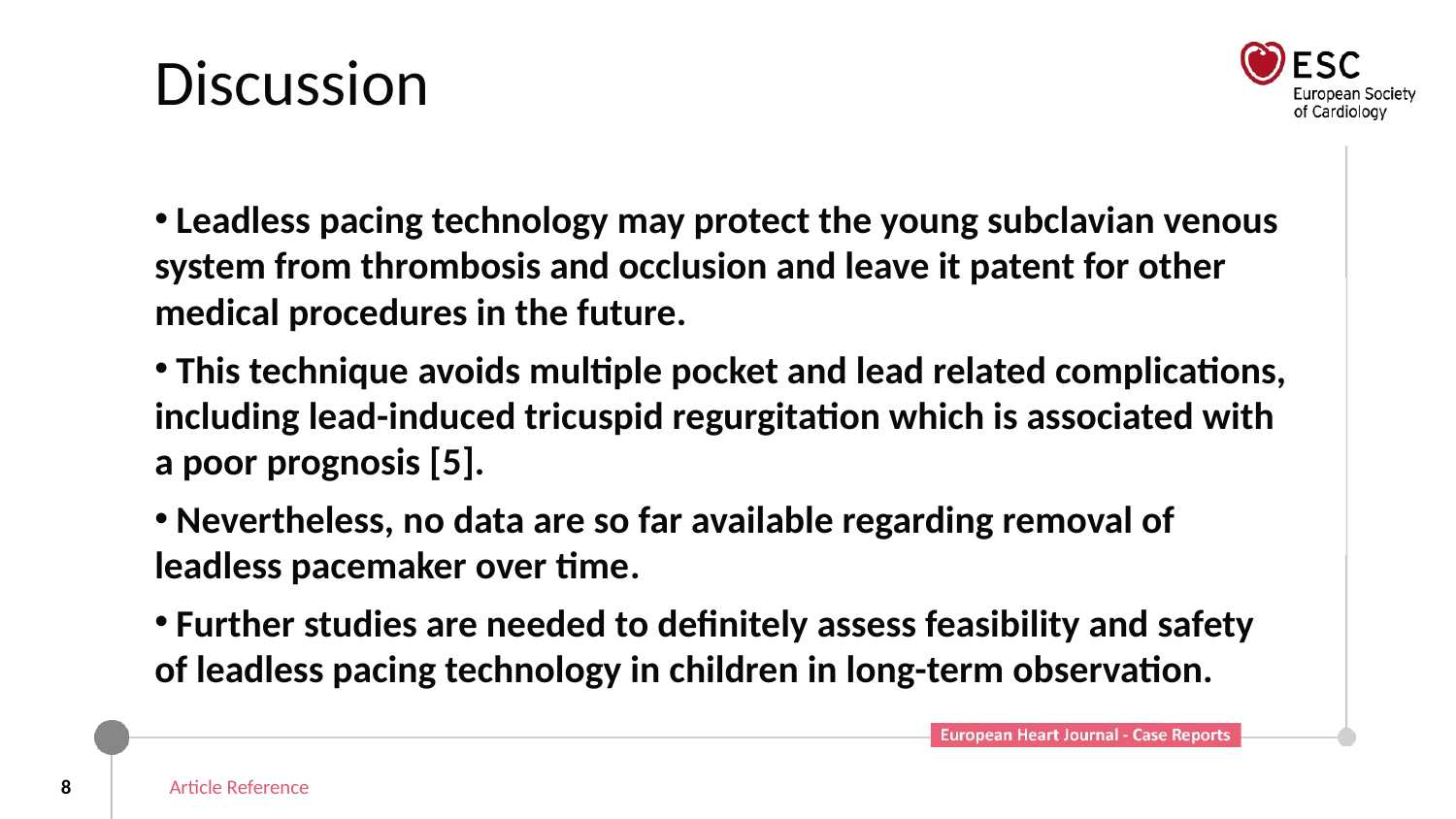

# Discussion
 Leadless pacing technology may protect the young subclavian venous system from thrombosis and occlusion and leave it patent for other medical procedures in the future.
 This technique avoids multiple pocket and lead related complications, including lead-induced tricuspid regurgitation which is associated with a poor prognosis [5].
 Nevertheless, no data are so far available regarding removal of leadless pacemaker over time.
 Further studies are needed to definitely assess feasibility and safety of leadless pacing technology in children in long-term observation.
8
Article Reference

## Slide 9
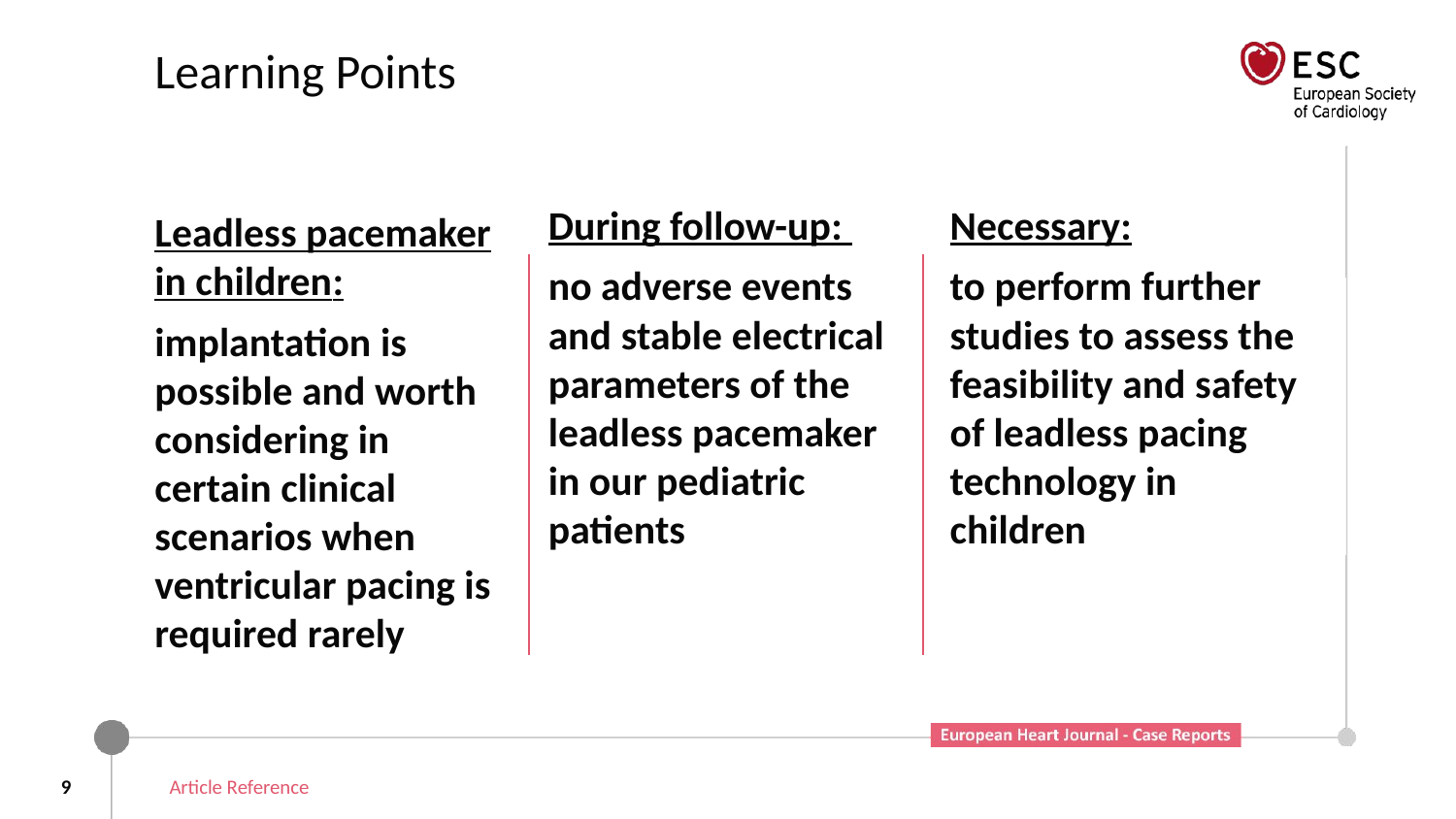

# Learning Points
During follow-up:
no adverse events and stable electrical parameters of the leadless pacemaker in our pediatric patients
Necessary:
to perform further studies to assess the feasibility and safety of leadless pacing technology in children
Leadless pacemaker in children:
implantation is possible and worth considering in certain clinical scenarios when ventricular pacing is required rarely
9
Article Reference

## Slide 10
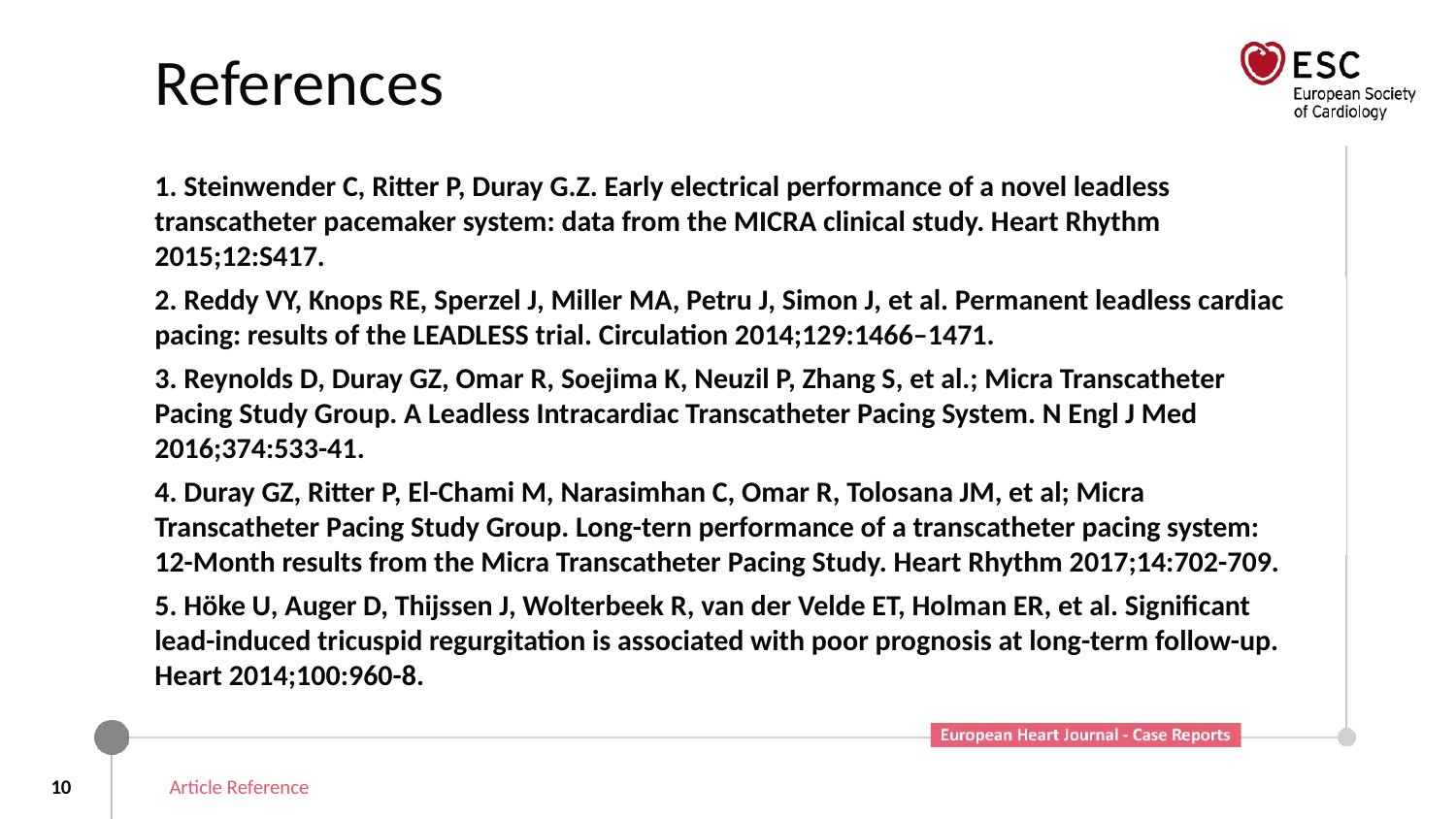

# References
1. Steinwender C, Ritter P, Duray G.Z. Early electrical performance of a novel leadless transcatheter pacemaker system: data from the MICRA clinical study. Heart Rhythm 2015;12:S417.
2. Reddy VY, Knops RE, Sperzel J, Miller MA, Petru J, Simon J, et al. Permanent leadless cardiac pacing: results of the LEADLESS trial. Circulation 2014;129:1466–1471.
3. Reynolds D, Duray GZ, Omar R, Soejima K, Neuzil P, Zhang S, et al.; Micra Transcatheter Pacing Study Group. A Leadless Intracardiac Transcatheter Pacing System. N Engl J Med 2016;374:533-41.
4. Duray GZ, Ritter P, El-Chami M, Narasimhan C, Omar R, Tolosana JM, et al; Micra Transcatheter Pacing Study Group. Long-tern performance of a transcatheter pacing system: 12-Month results from the Micra Transcatheter Pacing Study. Heart Rhythm 2017;14:702-709.
5. Höke U, Auger D, Thijssen J, Wolterbeek R, van der Velde ET, Holman ER, et al. Significant lead-induced tricuspid regurgitation is associated with poor prognosis at long-term follow-up. Heart 2014;100:960-8.
10
Article Reference
